# The use of post-cycle therapy is associated with reduced withdrawal symptoms from anabolic-androgenic steroid use: a survey of 470 men

**DOI:** 10.1186/s13011-023-00573-8

**Published:** 2023-11-11

**Authors:** Bonnie Grant, Joseph Kean, Naim Vali, John Campbell, Lorraine Maden, Prun Bijral, Waljit S. Dhillo, James McVeigh, Richard Quinton, Channa N. Jayasena

**Affiliations:** 1 Section of Investigative Medicine, Commonwealth Building, Imperial College London, Hammersmith Hospital, Du Cane Road, London, W12 0NN UK; 2 Bradford Metropolitan District Council, Britannia House, Hall Ings, Bradford, BD1 1HX UK; 3 Change Grow Live, London, UK; 4 Glasgow Alcohol & Drug Recovery Services, NHS Greater Glasgow & Clyde, Glasgow, Scotland, UK; 5 We Are With You, London, UK; 6 Department of Sociology, Manchester Metropolitan University, 4 Rosamund Street West, Manchester, M15 6LL UK; 7 Department of Endocrinology, Diabetes & Metabolism, Newcastle-Upon-Tyne Hospitals NHS Foundation Trust & Translational & Clinical Research Institute, University of Newcastle-Upon-Tyne, Newcastle, UK

**Keywords:** Anabolic–androgenic steroids, Hypogonadism, Image and performance enhancing drugs, Post-cycle therapy, Testosterone, Withdrawal

## Abstract

**Background:**

Anabolic–androgenic steroids (AAS) mimic the effects of testosterone and may include testosterone itself; they are used for body enhancement within the general population. AAS use has been linked with increased mortality, cardiovascular disease, mental health disorders, and infertility. AAS-induced hypogonadism can persist for an uncertain time period despite cessation, during which men may report physical and neuropsychiatric symptoms. In an attempt to mitigate these symptoms and expedite testicular recovery, many men self-administer post-cycle-therapy (PCT), typically involving human chorionic gonadotrophin (hCG) and selective oestrogen receptor modulators (SERMs), which are known to potently stimulate testicular function. However, this practice has no objective evidence of effectiveness to lessen the severity or duration of hypogonadal symptoms.

**Methods:**

An anonymous survey of four-hundred-and-seventy men using AAS explored the symptoms they experienced when ceasing AAS use; the effect of PCT on relieving their symptoms, and their perceived role for health service support.

**Results:**

The majority of respondents were white, aged 18–30 years old, and working in skilled manual work. 51.7% (*n* = 243) reported no issues with AAS use, but 35.3% reported increased aggression. 65.1% (*n* = 306) of respondents had attempted AAS cessation and 95.1% of these experienced at least one symptom upon AAS cessation. Low mood, tiredness and reduced libido were reported in 72.9%, 58.5% and 57.0% of men stopping AAS use, respectively, with only 4.9% reporting no symptoms. PCT had been used by 56.5% of respondents with AAS cessation and mitigated cravings to restart AAS use, withdrawal symptoms and suicidal thoughts by 60%, 60% and 50%, respectively. The effect of stopping AAS on body composition and recovery of testosterone or fertility was a concern in 60.5% and 52.4%, respectively. Most respondents felt PCT should be prescribed under medical supervision in the community.

**Conclusions:**

Our survey suggests that the majority of men stopping AAS use are using some form of PCT. Some self-reported symptoms of AAS-induced hypogonadism such as cravings to restart AAS use reduce by 60% and suicidal thoughts reduce by 50%. These individuals are concerned about the negative effect of AAS use and cessation. This study provides crucial information for planning future research to evaluate the effects of PCT on symptoms when men stop AAS use.

**Supplementary Information:**

The online version contains supplementary material available at 10.1186/s13011-023-00573-8.

## Introduction

Anabolic–androgenic steroids (AAS) are synthetic drugs that mimic the effects of testosterone. They are misused to promote aesthetic body enhancement, energy and improved well-being. AAS use was previously confined to improving physical performance in professional sporting and bodybuilding communities, but the practice has risen substantially within the general population [1–3]. Current best estimates predict that 2% of the U.K male population have used non-medically prescribed androgens, although the true figure may be even higher [4, 5]. AAS use has been linked with an increased mortality and risks of cardiovascular disease, transmission of blood-borne viruses, infertility, and mental health disorders [6–9].

AAS use suppresses endogenous luteinising hormone (LH)-mediated testosterone production, which may persist for many months to years after cessation [7, 10–12]. Men experiencing AAS-induced central hypogonadism may report physical and neuropsychiatric symptoms, including weakness, reduced libido, erectile dysfunction, depression, anxiety, and suicidality [10, 11, 13, 14]. Additionally, quality of life scores are found to be lower in men with all-cause hypogonadism, regardless of underlying pathology [15]. Some men resume AAS use to avoid experiencing these symptoms and thus enter a potentially dangerous cycle of dependence [16, 17].

Men using AAS may use different strategies to avoid symptoms of hypogonadism. Some may take a “blast and cruise” approach whereby large doses of AAS are used (for instance in preparation for an event) followed by a longer period of continued lower dose AAS. An alternative is “cycling”, whereby men use AAS for 6–12 weeks “on-cycle” followed by a period “off-cycle” when they take post-cycle therapy (PCT) [2, 18]. PCT is a non-medical term used to describe a varied group of self-administered substances, either with the aim to limit the adverse effects of AAS use between AAS cycles or to accelerate recovery of endogenous hypothalamic-pituitary–gonadal (HPG) axis function after permanent AAS cessation [3, 19, 20]. Various PCT regimes have been reported but typically involve the use of human chorionic gonadotrophin (hCG) in combination with selective oestrogen receptor modulators (SERM) and/or aromatase inhibitors (AI) [18, 19, 21, 22]. hCG directly stimulates testicular testosterone production within Leydig cells, while SERMs and AIs indirectly stimulate LH-mediated testosterone production and follicle-stimulating hormone (FSH)-mediated maintenance of seminiferous tubule mass by reducing the oestrogenic negative feedback on the HPG axis. By restoring endogenous testosterone production quicker than would have otherwise occurred, men hope to minimise or avert the symptoms of AAS-induced hypogonadism and thus avoid some of the negative health effects of AAS use. The use of PCT to restore HPG function after AAS cessation is unproven with no randomised controlled trials to support this approach.

There is currently minimal evidence describing the effect of PCT on symptoms of AAS-induced hypogonadism. We conducted a survey of 470 men using AAS to investigate their experiences when ceasing AAS use; the effect of PCT on symptom relief and to establish whether they perceive a role for health service support.

## Methods

An anonymous, self-administered, survey was completed between December 2021 and February 2023. Participants either currently or previously used AAS. The survey was distributed and completed online via Google Forms webpage or paper versions which were subsequently entered onto Google Forms by the study team. Individuals were recruited through adverts on websites related to AAS use, snowball sampling, and via practitioners working within harm reduction clinics and / or alongside drug treatment services that provide support for individuals using AAS in the U.K [23]. This was a survey evaluation as part of patient and public involvement for a research grant proposal and, therefore, does not require research ethical approval.

Participation in the survey was voluntary with participation implying informed consent. Information explaining the purpose of the study and assurance that all responses would be kept confidential was stated at the beginning of the survey. Questions covered demographic details such as age, ethnic group, location and occupation as defined by the U.K. Office for National Statistics [24]. Participants were also asked symptoms experienced when using and stopping AAS and PCT. No identifying data was collected. The survey consisted of up to 15 items of multiple-choice questions or Likert scales. Several questions allowed the respondent to select multiple applicable responses (Additional file 1).

### Data analysis

Completed questionnaires were submitted electronically to Google Forms and then tabulated using Microsoft Excel. Graphical techniques and analysis were performed using GraphPad Prism version 9. A total of 470 responses were received. Where appropriate, data are presented as median with interquartile range. Chi-squared tests were used to compare between groups larger than two. For all statistical testing, a *p* value of < 0.5 was considered significant.

## Results

### Demographics

The survey was completed 470 times by different individuals. 41.8% (*n* = 196) and 39.9% (*n* = 187) respondents were aged 18–30 years and 31–44 years respectively. The most common ethnicity was white (*n* = 298, 66.1%) and 62.2% (*n* = 291) lived in Yorkshire. Most respondents worked in skilled manual occupations (*n* = 129, 29.5%), followed by supervisory, clerical, and junior managerial (*n* = 80, 18.3%) and intermediate managerial roles (*n* = 72, 16.4%; Table 1).

**Table 1:** Demographics of survey respondents using anabolic–androgenic steroids

**Age (Years)**	**Respondents, *n* = 469 (%)**
18–30	196 (41.8)
31–44	187 (39.9)
45–60	81 (17.5)
60 +	4 (0.9)
**Ethnicity**	**Respondents, *n* = 470 (%)**
Asian or Asian British	100 (22.2)
Black, African, Caribbean, or Black British	18 (4.0)
Mixed or multiple ethnic groups	52 (11.5)
Other	2 (0.4)
White	298 (66.1)
**Location**	**Respondents, *n* = 468 (%)**
England: East	4 (0.9)
England: London and South-East	9 (1.0)
England: Midlands	14 (3.0)
England: North-East	17 (3.6)
England: North-West	14 (3.0)
England: South-West	43 (9.2)
England: Yorkshire	291 (62.2)
Scotland	57 (12.2)
Wales	13 (2.8)
Outside the UK	3 (0.6)
Other	3 (0.6)
**Occupation**	**Respondents, *n* = 438 (%)**
High-level managerial, administrative, or professional	30 (6.8)
Intermediate managerial, administrative, or professional	72 (16.4)
Supervisory, clerical, and junior managerial, administrative, or professional	80 (18.3)
Skilled manual work	129 (29.5)
Semi and unskilled manual work	67 (15.3)
Casual or lowest grade of work, unemployed or pension	42 (9.6)
Full-time student	18 (4.1)

### Adverse effects of anabolic–androgenic steroid use

Most (*n* = 227, 51.7%) survey respondents reported no problems with the use of AAS. 35.3% (*n* = 155) reported increased aggression, 8.0% (*n* = 35) violence and 1.4% (*n* = 6) reported imprisonment that they associated with their AAS use. Respondents were able to report additional problems experienced with AAS use as a free text as “other”. Testicular atrophy, acne and mood swings were the most commonly reported additional problems (10.0%, 4.3% and 4.1% respectively; Table 2).

**Table 2:** Reported problems with anabolic–androgenic steroid use by survey respondents

Problems reported with anabolic–androgenic steroid use	Respondents, *n* = 439 (%)
No problems	227 (51.7)
Becoming more aggressive than usual	155 (35.3)
Becoming violent	35 (8.0)
Prison	6 (1.4)
Other:	
Testicular atrophy	44 (10.0)
Gynaecomastia	10 (2.3)
Acne	19 (4.3)
Mood swings	18 (4.1)
Changes in hair growth	15 (3.4)
Increased sex drive	9 (2.1)

### Anabolic–androgenic steroid cessation and post-cycle therapy

**Fig. 1:**
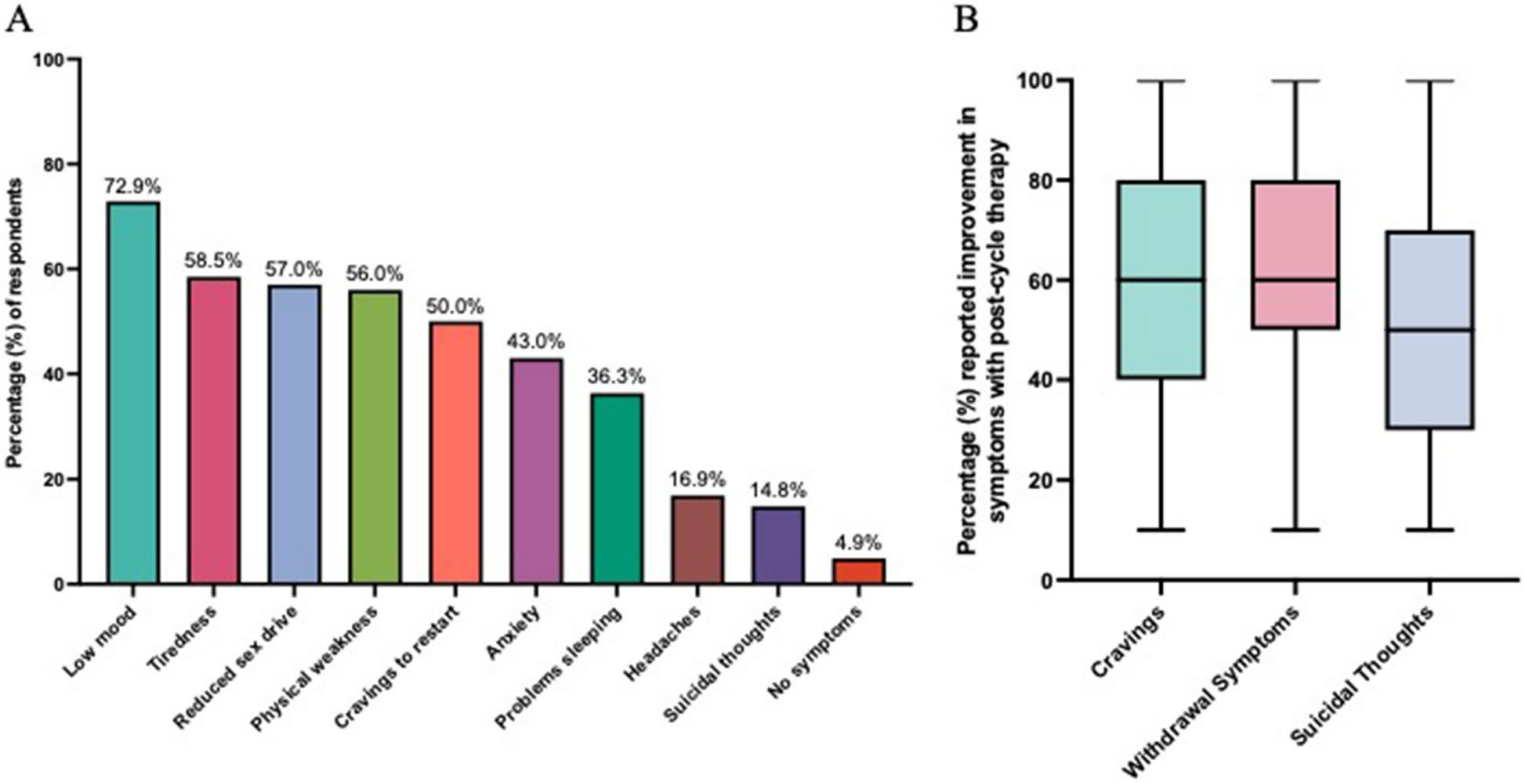
**A** Percentage (%) of respondents attempting anabolic–androgenic steroid cessation experiencing anabolic–androgenic steroid induced hypogonadal symptoms. Total *n* = 284 respondents. **B** Percentage (%) reported improvement in symptoms with respondents using post-cycle therapy. Data is shown for cravings/urge to restart anabolic–androgenic steroid use (*n* = 158), withdrawal symptoms (*n* = 105) and suicidal thoughts (*n* = 27). Data is presented as median, interquartile range, minimum and maximum. Scale is 10 – 100% with 100% being the maximum improvement

An attempt at AAS cessation was reported by 65.1% (*n* = 306) of all respondents. 95.1% of respondents who had attempted AAS cessation reported at least one symptom upon AAS cessation. Low mood, tiredness, reduced sex drive and physical weakness was reported in over fifty percent of respondents who had attempted AAS cessation (72.9%, 58.5%, 57.0% and 56.0% respectively; Fig. 1A). Fourteen respondents (4.9%) reported no symptoms with AAS cessation.

PCT use when stopping AAS was reported in 56.5% (*n* = 160) of respondents. Within our anonymous survey, PCT use was self-reported to reduce cravings to restart AAS by 60% (IQR 40—80), withdrawal symptoms by 60% (IQR 50 – 80) and suicidal thoughts by 50% (IQR 30 – 70; Fig. 1B).

58.1% (*n* = 273) respondents felt it was unlikely, or very unlikely they would stop AAS use in the next 5 years. The effects of stopping AAS on body composition or physical performance and the uncertain recovery of testosterone or fertility were reported as the biggest concerns in relation to stopping AAS (*n* = 268; 60.5% and *n* = 232; 52.4% respectively). Effectiveness and purity of PCT drugs were a concern for 41.1% (*n* = 182), while 24.8% and 24.2% were concerned about quality and access of NHS advice respectively. 31.0% (*n* = 145) were interested, or very interested in participating in future trials of PCT. The majority of respondents (*n* = 206; 43.8%) felt that the community was the best place for NHS prescribed PCT, while only 10.0% (*n* = 47) preferred NHS specialist clinics (Table 3).

**Table 3:** Anabolic–androgenic steroid cessation and post-cycle therapy

**Likelihood of stopping anabolic–androgenic steroids in next 5 years**	**Respondents, *n* = 470 (%)**
1 (Very unlikely)	135 (28.7)
2	138 (29.4)
3	96 (20.4)
4	48 (10.2)
5 (Very likely)	53 (11.3)
**Worries about stopping anabolic–androgenic steroids**	**Respondents, *n* = 443 (%)**
Nothing	82 (18.5)
Recovery of testosterone or fertility	232 (52.4)
Effects on body composition or physical performance	268 (60.5)
Access to NHS for advice	107 (24.2)
Quality of NHS advice	110 (24.8)
Effectiveness or purity of PCT	182 (41.1)
Other	20 (4.5)
**Interest in participating in a research trial about post-cycle therapy**	**Respondents, *n* = 469 (%)**
1 (Not at all interested)	156 (33.3)
2	76 (16.2)
3	92 (19.6)
4	42 (9.0)
5 (Very interested)	103 (22.0)
**Where would be best to access National Health Service (NHS) prescribed post-cycle therapy?**	**Respondents, *n* = 470 (%)**
Community e.g., harm prevention clinic or local pharmacy	206 (43.8)
GP surgery	109 (23.2)
NHS Specialist clinic e.g., endocrinology	47 (10.0)
Online service	138 (29.4)

### Association with age

The association between age groups and survey responses are shown in Additional file 2. Older men had significantly higher rates of attempted steroid cessation compared with younger men. Increasing age was associated with greater self-reporting low mood (*p* = 0.0001), problems sleeping (*p* = 0.0011) and headache (*p* = 0.0007) when stopping AAS use. Younger men were less likely to report problems with AAS use (*p* < 0.001) and had fewer worries about stopping AAS use (*p* < 0.0001). Younger men preferred PCT to be available online (*p* < 0.0001) whereas older men preferred from general practitioners (*p* = 0.0038).

## Discussion

Anabolic–androgenic steroid use has risen within the general population, and many use PCT as an unproven practice to avoid or minimise the negative health effects associated with AAS use and cessation. There are currently no clinical guidelines for managing AAS-induced hypogonadism, and men often seek substances and advice from internet sources and peers, rarely health care professionals [2, 25, 26]. This study provides several novel insights into experiences of AAS cessation and the role of PCT on symptoms.

The demographics within our study are broadly similar to previously reported [2, 3, 19]. Respondents were commonly aged 18–44 years old, white ethnicity, and working in skilled manual jobs. They were disproportionately from Yorkshire, the largest county in Northern England. The majority of respondents reported no adverse effects with the use of AAS, contrasting with higher rates reported in other studies [3, 19, 20, 27]. This may be due to the limited response options given to our participants, or a reluctance to divulge this information. A recent meta-analysis estimated that 73% of AAS found on the black market were counterfeit or of substandard quality; this may explain why half of our respondents did not report any adverse effects with AAS use [28]. The most reported symptoms upon cessation in our survey were low mood, reduced libido, tiredness, and physical weakness have been reported previously [10, 11, 14].

Fifty-seven percent of respondents self-reported reduced libido when stopping AAS use. Two studies have reported improvements in erectile dysfunction (ED) symptoms with drugs commonly used as part of PCT. Firstly, men with a history of non-prescribed androgen use were recruited to a single-centre cohort study to receive treatment with hCG and clomiphene versus observation, based on patient choice [29]. Following AAS cessation, there was no statistical significance between the two groups International Index of Erectile Function (IIEF) scores. In those who did not receive treatment, a statistically significant improvement in IIEF scores was not observed until 12 months after recruitment, whereas in the treatment group, scores improved from 6 months onward. A major limitations was that participants were given the choice of intervention or observation, and so the conclusions drawn may be due to placebo effect. Secondly, a cross-sectional observational study by Armstrong et al. assessed sexual function of 231 men currently, or previously using AAS with the abbreviated 5-item International Index of Erectile Function (IIEF-5) [30]. They reported higher IIEF-5 scores in those who concurrently used other substances, such as anti-oestrogens suggesting this may be a protective factor in maintaining erectile function after AAS use. While these two studies show higher IIEF scores with hCG and SERM use, the lack of randomisation or placebo-control limits their conclusions.

There are currently no studies that quantitatively evaluate the effect of PCT on neuropsychiatric symptoms. In our anonymous survey, the use of self-administered PCT reduced self-reported craving symptoms and withdrawal symptoms by 60% each and suicidal thoughts by 50%. Griffiths et al.’s thematic analysis reported increased mood disturbances upon AAS cessation with some reporting an improvement in symptoms with PCT [31]. Others however reported that the use of PCT worsened some psychiatric symptoms. Additionally, difficulty accessing PCT during the COVID-19 pandemic has been tentatively linked to worsening of mental health disorders in men ceasing AAS use, however this was likely multicausal [32]. While some of the improved symptoms reported in the survey may be explained by placebo effect, there may be a role for PCT in managing symptoms of AAS-induced hypogonadism.

While the majority of respondents felt it was unlikely or very unlikely they would stop AAS use in the next five years, this may be explained by worries around the effects of AAS cessation. Our findings showed the effect of AAS cessation on body composition or physical performance and recovery of testosterone levels or fertility was of concern to respondents. These findings are corroborated by Griffiths et al. who reported participants awareness of the long term health consequences of AAS use, and PCT was a means used to maintain health or gains, of which fertility risk was a key concern [31]. Participants also reported challenges in obtaining PCT; in fact, accessing AAS was deemed much easier, which led some to prolong or indefinitely continue AAS use. Our findings found that 41.1% of respondents were concerned about the effectiveness or purity of PCT. Most PCT is obtained illicitly through online sources or peers and so the verification of active hCG or SERM substances within these products often cannot be confirmed [28]. Piatkowski et al. also reported on concerns about the legitimacy of AAS or PCT substances used. The men interviewed felt that if the legitimacy of substances could be confirmed it would encourage safer practices and reduce potentially dangerous adverse effects [33]. The concerns highlighted in this study may be preventing cessation in many men and are therefore a target for cessation programmes. Importantly, there was an expression of interest in participating in a research trial about PCT by some of the respondents and engagement from within the community is vital for any future intervention studies. Around a quarter of respondents to this survey were concerned about access to NHS advice or quality of NHS advice. Many physicians have attempted to manage a patient with AAS-induced hypogonadism, but most do not feel confident in treating the condition [34]. Patients are often advised to wait until symptoms resolve, with many continuing to experience unresolved symptoms. Physicians are often rated poorly by men using AAS regarding their knowledge and expertise when compared with coaches, bodybuilding websites and other men using AAS [3, 26]. In the U.K., men using AAS may seek support through community harm reduction clinics [23]. The availability, and trust, in this support network may explain why most respondents would prefer to access prescribed PCT in the community, rather than via specialist clinics or their general practitioner.

The drugs used as PCT are commonly used in the management of male hypogonadism, however there are no current controlled trials demonstrating their efficacy or safety in AAS-induced hypogonadism [35–37]. It is therefore premature to recommend its use to healthcare professionals. If PCT was objectively shown to help men with AAS-induced hypogonadism, engagement with healthcare professionals using interviews and focus groups to scope their attitudes would be important to translate PCT into practice.

Despite the novel aspects identified in our study there are several limitations. 60% of respondents were from the Yorkshire area and our findings may reflect regional variations in practice, particularly around PCT use. It is unlikely this has significantly changed our conclusions however as the proportion of respondents using PCT in the non-Yorkshire area was similar to the whole group. As the survey was voluntary and with a focus on PCT, this exposes selection bias to those using PCT and, as it required respondents to recall their previous experiences, it is necessarily subject to recall bias and uncertainty. PCT describes a heterogeneous group of drugs and differing regimes are often used. A limitation of our study is that no information was collected about duration or specific drugs used as PCT, which may have helped identify specific patterns of usage associated with most experienced benefit. Details about prior history of AAS use, dosage and duration were not collected which may influence reported symptoms. This may have aided with interpretation of symptoms experienced by respondents to our survey. Finally, the self-reported improvement in symptoms may also be attributable to a placebo effect, and not a direct PCT effect.

In summary, our study provides novel insights into the experience of AAS cessation and the perceived benefit of PCT on symptoms of AAS-induced hypogonadism. Respondents of our anonymous survey self-reported physical and neuropsychiatric symptoms of hypogonadism which improved with self-administered PCT use. The information from this study is critical for firstly planning future well-designed randomised controlled studies to compare the effectiveness of cessation versus hormonal treatment in managing AAS-induced hypogonadism. Once established, any successful treatment is likely to require an integrated approach engaging healthcare professionals and peer networks to address the endocrine and neuropsychiatric symptoms experienced by men motivated to stop AAS use long-term.

### Supplementary Information

**Additional file 1:** Complete survey administered to participants.

**Additional file 2: **Supplemental Table 1: Survey responses by age group.

## Data Availability

The datasets used and analysed during the current study are available from the corresponding author on reasonable request.

## Abbreviations

AAS: Anabolic–androgenic steroids
AI: Aromatase inhibitors
FSH: Follicle-stimulating hormone
hCG: Human chorionic gonadotrophin
HPG: Hypothalamic-pituitary–gonadal
IIEF: International Index of Erectile Function
IQR: Interquartile range
LH: Luteinising hormone
NHS: National Health Service
PCT: Post-cycle therapy
SERM: Selective oestrogen receptor modulators

## References

[1] Piatkowski TM, White KM, Hides LM, Obst PL (2020). Australia’s Adonis: understanding what motivates young men’s lifestyle choices for enhancing their appearance. Australian Psychol.

[2] Cohen J, Collins R, Darkes J, Gwartney D (2007). A league of their own: demographics, motivations and patterns of use of 1,955 male adult non-medical anabolic steroid users in the United States. J Int Soc Sports Nutr.

[3] Bonnecaze AK, O’Connor T, Aloi JA (2020). Characteristics and attitudes of men using anabolic androgenic steroids (AAS): a Survey of 2385 men. Am J Mens Health.

[4] Hope VD, Walker Bond V, Boardley I, Smith J, Campbell J, Bates G, et al. Anabolic androgenic steroid use population size estimation: a first stage study utilising a Delphi exercise. Drugs: Education, Prevention and Policy. 2023;30(5):461-73.

[5] Mullen C, Whalley BJ, Schifano F, Baker JS (2020). Anabolic androgenic steroid abuse in the United Kingdom: an update. Br J Pharmacol.

[6] Horwitz H, Andersen JT, Dalhoff KP (2019). Health consequences of androgenic anabolic steroid use. J Intern Med.

[7] Shankara-Narayana N, Yu C, Savkovic S, Desai R, Fennell C, Turner L (2020). Rate and extent of recovery from reproductive and cardiac dysfunction due to androgen abuse in men. J Clin Endocrinol Metab..

[8] Pope HG, Katz DL (1994). Psychiatric and medical effects of anabolic-androgenic steroid use. A controlled study of 160 athletes. Arch Gen Psychiatry.

[9] Hope VD, McVeigh J, Marongiu A, Evans-Brown M, Smith J, Kimergård A (2013). Prevalence of, and risk factors for, HIV, hepatitis B and C infections among men who inject image and performance enhancing drugs: a cross-sectional study. BMJ Open.

[10] Kanayama G, Hudson JI, DeLuca J, Isaacs S, Baggish A, Weiner R (2015). Prolonged hypogonadism in males following withdrawal from anabolic-androgenic steroids: an under-recognized problem. Addiction.

[11] Rasmussen JJ, Selmer C, Østergren PB, Pedersen KB, Schou M, Gustafsson F (2016). Former abusers of anabolic androgenic steroids exhibit decreased testosterone levels and hypogonadal symptoms years after cessation: a case-control study. PLoS One.

[12] Smit DL, Buijs MM, de Hon O, den Heijer M, de Ronde W (2021). Disruption and recovery of testicular function during and after androgen abuse: the HAARLEM study. Hum Reprod.

[13] Pope HG, Katz DL (1988). Affective and psychotic symptoms associated with anabolic steroid use. Am J Psychiatry.

[14] Sharma A, Grant B, Islam H, Kapoor A, Pradeep A, Jayasena CN (2022). Common symptoms associated with usage and cessation of anabolic androgenic steroids in men. Best Pract Res Clin Endocrinol Metab.

[15] Zitzmann M (2020). Testosterone, mood, behaviour and quality of life. Andrology.

[16] Ip EJ, Lu DH, Barnett MJ, Tenerowicz MJ, Vo JC, Perry PJ (2012). Psychological and physical impact of anabolic-androgenic steroid dependence. Pharmacotherapy.

[17] Kanayama G, Brower KJ, Wood RI, Hudson JI, Pope HG (2009). Jr. Anabolic-androgenic steroid dependence: an emerging disorder. Addiction.

[18] Karavolos S, Reynolds M, Panagiotopoulou N, McEleny K, Scally M, Quinton R (2015). Male central hypogonadism secondary to exogenous androgens: a review of the drugs and protocols highlighted by the online community of users for prevention and/or mitigation of adverse effects. Clin Endocrinol (Oxf).

[19] Smit DL, de Hon O, Venhuis BJ, den Heijer M, de Ronde W (2020). Baseline characteristics of the HAARLEM study: 100 male amateur athletes using anabolic androgenic steroids. Scand J Med Sci Sports.

[20] Parkinson AB, Evans NA (2006). Anabolic androgenic steroids: a survey of 500 users. Med Sci Sports Exerc.

[21] de Ronde W, Smit DL (2020). Anabolic androgenic steroid abuse in young males. Endocr Connect.

[22] Rochoy M, Danel A, Chazard E, Gautier S, Berkhout C (2022). Doping with aromatase inhibitors and oestrogen receptor modulators in steroid users: analysis of a forum to identify dosages, durations and adverse drug reactions. Therapie.

[23] Kimergård A, McVeigh J (2014). Variability and dilemmas in harm reduction for anabolic steroid users in the UK: a multi-area interview study. Harm Reduct J.

[24] Statistics ON (2023). Census 2021.

[25] Amaral JMX, Kimergard A, Deluca P (2022). Prevalence of anabolic steroid users seeking support from physicians: a systematic review and meta-analysis. BMJ Open.

[26] Pope HG, Kanayama G, Ionescu-Pioggia M, Hudson JI (2004). Anabolic steroid users’ attitudes towards physicians. Addiction.

[27] Rowe R, Berger I, Copeland J (2017). No pain, no gainz? Performance and image-enhancing drugs, health effects and information seeking. Drugs: Educ Prev Policy.

[28] Magnolini R, Falcato L, Cremonesi A, Schori D, Bruggmann P (2022). Fake anabolic androgenic steroids on the black market - a systematic review and meta-analysis on qualitative and quantitative analytical results found within the literature. BMC Public Health.

[29] Al Hashimi M (2022). The deleterious effects of anabolic androgenic steroid abuse on sexual and reproductive health and comparison of recovery between treated and untreated patients: single-center prospective randomized study. Andrologia.

[30] Armstrong JM, Avant RA, Charchenko CM, Westerman ME, Ziegelmann MJ, Miest TS (2018). Impact of anabolic androgenic steroids on sexual function. Transl Androl Urol.

[31] Griffiths S, Henshaw R, McKay FH, Dunn M (2017). Post-cycle therapy for performance and image enhancing drug users: a qualitative investigation. Perform Enhancement Health.

[32] Dunn M, Piatkowski T (2021). Investigating the impact of COVID-19 on performance and image enhancing drug use. Harm Reduct J.

[33] Piatkowski T, Puljevic C, Francis C, Ferris J, Dunn M (2023). They sent it away for testing and it was all bunk: exploring perspectives on drug checking among steroid consumers in Queensland, Australia. Int J Drug Policy.

[34] Grant B, Pradeep A, Minhas S, Dhillo WS, Quinton R, Jayasena CN (2023). Survey of endocrinologists managing recovery from anabolic androgenic steroid induced hypogonadism. Reprod Fertil.

[35] Jayasena CN, Anderson RA, Llahana S, Barth JH, MacKenzie F, Wilkes S (2022). Society for endocrinology guidelines for testosterone replacement therapy in male hypogonadism. Clin Endocrinol (Oxf).

[36] Wenker EP, Dupree JM, Langille GM, Kovac J, Ramasamy R, Lamb D (2015). The use of HCG-Based combination therapy for recovery of spermatogenesis after testosterone use. J Sex Med.

[37] Huijben M, Lock M, de Kemp VF, de Kort LMO, van Breda HMK (2022). Clomiphene citrate for men with hypogonadism: a systematic review and meta-analysis. Andrology.

