# Supplementary material for: The use of post-cycle therapy is associated with reduced withdrawal symptoms from anabolic-androgenic steroid use: a survey of 470 men
Source: Subst Abuse Treat Prev Policy. 2023 Nov 11;18:66. doi: 10.1186/s13011-023-00573-8 (PMC10640727; doi:10.1186/s13011-023-00573-8)
Supplement: Supplementary file 1 — Additional file 1: Complete survey administered to participants. [file 13011_2023_573_MOESM1_ESM.docx]

**Appendix 1: Complete survey administered to participants.**

1. Have you ever tried stopping steroids?
□ YES □ NO **(if no, go to question 7)**

2. When you stopped taking steroids, which of these symptoms did you get? Tick all that apply

□ I had no symptoms □ Tiredness

□ Problems sleeping □ Suicidal thoughts

□ Headaches □ Physical weakness

□ Cravings to restart steroids □ Reduced sex drive

□ Low Mood □ Anxiety

□ Other: ____________________

3. Did you take PCT when you stopped steroids?

□ YES □ NO **(if no, go to question 7)**

4. When you stopped taking steroids, how much did PCT reduce your craving (urge to restart steroids)? (0 to 100%)

1 2 3 4 5 6 7 8 9 10

0% □ □ □ □ □ □ □ □ □ □ 100%

5. When you stopped taking steroids, how much did PCT reduce your withdrawal symptoms (feeling bad from stopping steroids)? (0 to 100%)

1 2 3 4 5 6 7 8 9 10

0% □ □ □ □ □ □ □ □ □ □ 100%

6. ONLY ANSWER IF YOU HAD SUICIDAL THOUGHTS WHEN STOPPING STEROIDS:

Did PCT reduce your suicidal thoughts?

1 2 3 4 5 6 7 8 9 10

0% □ □ □ □ □ □ □ □ □ □ 100%

7. How likely are you to stop steroids in the next 5 years?

1 2 3 4 5

Very unlikely □ □ □ □ □ Very likely

8. Did steroids give you any of these problems? Tick all that apply

□ No problems

□ Becoming more aggressive than usual

□ Becoming violent

□ Prison

□ Other: ____________________

9. Do any of these worry you about stopping steroids? Tick all that apply

□ Nothing worries me about stopping

□ Recovery of testosterone levels or fertility

□ Effects on body shape or physical performance

□ Access to NHS advice about stopping

□ Quality of NHS advice about stopping

□ Effectiveness, safe or purity of PCT drugs

□ Other: ____________________

10. We want to do a clinical trial to find out which PCT protocol (mix of hormones like hCG, clomiphene) gives the best results. How interested are you in participating?

1 2 3 4 5

Not at all interested □ □ □ □ □ Very interested

11. Where would be best to access NHS prescribed PCT?

□ Community e.g. harm prevention clinic or local pharmacy

□ GP surgery

□ NHS specialist clinic e.g. endocrinology

□ Online service

12. What is your age?

□ Under 18 □ 18-30 □ 31-44 □ 45-60 □ Over 60

13. What is your ethnic group?

□ White □ Asian or Asian British

□ Mixed or Multiple ethnic groups □ Other ethnic group

□ Black, African, Caribbean or Black British

14. What is your type of occupation?

□ High-level managerial, administrative or professional

□ Intermediate managerial, administrative or professional

□ Supervisory, clerical and junior managerial, administrative or professional

□ Skilled manual work

□ Semi and unskilled manual work

□ Casual or lowest grade of work, unemployed or pension

□ Full-time student

15. Where do you live?

□ England: London and South-East □ Yorkshire

□ England: South West □ Scotland

□ England: East □ Wales

□ England: Midlands □ Northern Ireland

□ England: North-East □ Outside the UK

□ England: North-West
