# Supplementary material for: The use of post-cycle therapy is associated with reduced withdrawal symptoms from anabolic-androgenic steroid use: a survey of 470 men
Source: Subst Abuse Treat Prev Policy. 2023 Nov 11;18:66. doi: 10.1186/s13011-023-00573-8 (PMC10640727; doi:10.1186/s13011-023-00573-8)
Supplement: Supplementary file 2 — Additional file 2: Supplemental Table 1: Survey responses by age group. [file 13011_2023_573_MOESM2_ESM.docx]

**Supplemental Table 1: Survey responses by age group**

|  | **Age (Years)** | | | **P Value** |
| --- | --- | --- | --- | --- |
|  | **18 – 30**  **n (%)** | **31 – 44**  **n (%)** | **45 +**  **n (%)** |  |
| **Problems reported with anabolic-androgenic steroid use** | | | | |
| **No problems** | 116 (64.4) | 77 (43.3) | 34 (42.5) | <0.001 |
| **Becoming more aggressive than usual** | 38 (21.1) | 75 (42.1) | 41 (51.3) | <0.001 |
| **Becoming violent** | 5 (2.8) | 21 (11.8) | 8 (10.0) | 0.0044 |
| **Prison** | 1 (0.6) | 5 (2.8) | 0 (0) | 0.0943 |
| **Testicular atrophy** | 20 (11.1) | 21 (11.8) | 3 (3.8) | 0.1142 |
| **Gynaecomastia** | 5 (2.8) | 5 (2.8) | 0 (0) | 0.3187 |
| **Acne** | 10 (5.6) | 7 (3.9) | 2 (2.5) | 0.5054 |
| **Mood Swings** | 7 (3.9) | 8 (4.5) | 3 (3.8) | 0.9439 |
| **Hair Growth** | 6 (3.3) | 7 (3.9) | 2 (2.5) | 0.8394 |
| **Increased sex drive** | 2 (1.1) | 4 (2.2) | 3 (3.8) | 0.3732 |
|  | | | | |
| **Problems reported with anabolic-androgenic steroid cessation** | | | | |
| **No symptoms** | 2 (2.2) | 9 (6.1) | 3 (4.3) | 0.3842 |
| **Cravings to restart** | 38 (42.7) | 68 (46.3) | 35 (50.7) | 0.6042 |
| **Low mood** | 52 (58.4) | 61 (41.5) | 49 (71.0) | 0.0001 |
| **Anxiety** | 23 (25.8) | 66 (44.9) | 12 (17.4) | <0.001 |
| **Reduced sex drive** | 41 (46.1) | 77 (52.4) | 24 (34.8) | 0.0535 |
| **Tiredness** | 44 (49.4) | 78 (53.1) | 43 (62.3) | 0.2567 |
| **Suicidal thoughts** | 12 (13.5) | 20 (13.6) | 10 (14.5) | 0.9803 |
| **Problems sleeping** | 18 (20.2) | 52 (35.4) | 33 (47.8) | 0.0011 |
| **Headaches** | 11 (12.4) | 16 (10.9) | 21 (30.4) | 0.0007 |
| **Physical weakness** | 43 (48.3) | 74 (50.3) | 41 (59.4) | 0.3390 |
|  | | | | |
| **Used PCT when stopping steroids** | 42 (54.5) | 59 (42.1) | 37 (56.9) | 0.0737 |
|  | | | | |
| **Worries about stopping steroids** | | | | |
| **Nothing** | 56 (30.8) | 17 (9.4) | 9 (11.3) | <0.0001 |
| **Recovery of testosterone or fertility** | 74 (40.7) | 121 (67.2) | 36 (45.0) | <0.0001 |
| **Effect on body composition or physical performance** | 88 (48.4) | 125 (69.4) | 54 (67.5) | <0.0001 |
| **Access to NHS for advice** | 24 (13.2) | 53 (29.4) | 30 (37.5) | <0.0001 |
| **Quality of NHS advice** | 17 (9.3) | 59 (32.8) | 33 (41.3) | <0.0001 |
| **Effectiveness or purity of PCT** | 55 (30.2) | 82 (45.6) | 45 (56.3) | 0.0001 |
|  | | | | |
| **Where should PCT be available?** | | | | |
| **Community e.g., harm prevention clinic or local pharmacy** | 80 (40.8) | 86 (46.0) | 40 (46.5) | 0.5153 |
| **Online service** | 88 (44.9) | 38 (20.3) | 12 (14.0) | <0.0001 |
| **NHS Specialist Clinic e.g., endocrinology** | 8 (4.1) | 28 (15.0) | 9 (10.5) | 0.0014 |
| **General practitioner surgery** | 33 (16.8) | 43 (23.0) | 30 (34.9) | 0.0038 |
